# AKIP1, a Cardiac Hypertrophy Induced Protein that Stimulates Cardiomyocyte Growth via the Akt Pathway

**DOI:** 10.3390/ijms141121378

**Published:** 2013-10-28

**Authors:** Hongjuan Yu, Wardit Tigchelaar, Bo Lu, Wiek H. van Gilst, Rudolf A. de Boer, B. Daan Westenbrink, Herman H. W. Silljé

**Affiliations:** 1 Department of Cardiology, University Medical Center Groningen, University of Groningen, Hanzeplein 1, 9713 GZ Groningen, P.O. Box 30.001, Groningen 9700 RB, The Netherlands; E-Mails: h.yu@umcg.nl (H.Y.); w.tigchelaar@umcg.nl (W.T.); bolu80@gmail.com (B.L.); w.h.van.gilst@umcg.nl (W.H.G.); r.a.de.boer@umcg.nl (R.A.B.); b.d.westenbrink@umcg.nl (B.D.W.); 2 Department of Hematology, the First Affiliated Hospital of Harbin Medical University, 23 Youzheng Street, Nangang District, Harbin 150001, China

**Keywords:** cardiac hypertrophy, AKIP1, Akt, kinase, remodeling

## Abstract

Cardiac adaptation to unremitting physiological stress typically involves hypertrophic growth of cardiomyocytes, a compensatory response that often fails and causes heart disease. Gene array analysis identified *AKIP1* (A Kinase Interacting Protein 1) as a hypertrophic gene and we therefore hypothesized a potential role in the hypertrophic response. We show for the first time that both AKIP1 mRNA and protein levels increased in hypertrophic cardiomyocytes under conditions of sustained cardiac stress, including pressure overload and after myocardial infarction and *in vitro* in phenylephrine (PE) stimulated neonatal rat ventricular cardiomyocytes (NRVCs). AKIP1 overexpression in NRVCs markedly stimulated hypertrophic growth responses, including significantly increased cell size, augmented cytoskeletal organization and protein synthesis. Although, AKIP1 was not essential for PE induced hypertrophy in NRVCs, it did potentiate neurohormonal induced protein synthesis. AKIP1 did, however, not induce expression of pathological marker genes like ANP and β-MHC. ERK and Akt kinase signaling pathways have been linked to hypertrophy and AKIP1 specifically induced phosphorylation of Akt. This phosphorylation of Akt was essential for activation of ribosomal rpS6 and translation elongation factor eEF2 and this readily explains the increased protein synthesis. Akt inhibition fully blocked AKIP1 induced hypertrophy, showing that this pathway is critically involved. In conclusion, our results show that AKIP1 is induced in hypertrophic hearts and can stimulate adaptive cardiomyocyte growth, which involves Akt signaling.

## 1. Introduction

Cardiovascular diseases are a leading cause of morbidity and mortality in many countries. To maintain continuous blood flow and to fulfill the body demands, the heart is able to adapt to various conditions [1]. Adaption to stress conditions is essential, but the molecular mechanisms that contribute to physiological and pathological adaptations of the heart are complex and still poorly understood [2–4]. An important adaptive and compensatory response to cardiac stress is cardiomyocyte growth or hypertrophy [1]. This allows reduction of wall stress under conditions of pressure or volume overload that may arise from different causes. These include pathological factors like valve insufficiency, hypertension, or myocardial infarction, but also physiological triggers like exercise or pregnancy [1,4]. Although hypertrophy is essentially an adaptive response to cardiac stress, it may become maladaptive under conditions of sustained pathological stress. Under those conditions it can result in cardiac remodeling, which is considered to be one of the most important risk factors for cardiac morbidity and mortality [3,5]. It is therefore imperative to understand the molecular mechanisms contributing to hypertrophy development. Amongst others, Akt and ERK kinase signaling pathways have been shown to contribute to both physiological and pathological hypertrophy [6–9].

In a recent report a kinase interacting protein 1 (AKIP1, also termed BCA3) was identified as a potential new factor controlling stress adaptation in the heart [10]. In particular, it was shown that increased AKIP1 levels in the heart protected against ischemia/reperfusion and improved cardiac function. AKIP1 was found in a two-hybrid screen with protein kinase A (PKA) as a bait (hence its current name) [11], but in the meantime, interactions with multiple proteins have been reported, suggestive for an adaptor function of AKIP1 in signaling pathways [12–15]. Although its exact function still remains elusive most studies hint towards roles in signal transduction, cell proliferation and survival [11,15–17]. This is particularly true for cancer cell lines, in which AKIP1 has been primarily investigated and in which it was originally identified as a breast cancer expressed gene [18]. Functional studies in normal cells are scarce, but with the recent identification of AKIP1 as a protein involved in stress adaptation of the heart to ischemia/reperfusion, this is likely to change fast [10].

Using gene expression arrays, we recently identified a concise set of genes that were differentially regulated in multiple animal hypertrophy models. Interestingly, one of the identified genes that was prominently upregulated was *AKIP1* [19]. In humans, there are three splice variants, the full-length protein (AKIP1a), one that lacks the third exon (AKIP1b), and one that lacks the third and fifth exon (AKIP1c). In contrast, only the full-length protein is present in rodents [20]. Whether AKIP1 protein levels are upregulated under these stress conditions and whether this has functional consequences is, however, unknown. We therefore investigated the potential involvement of AKIP1 in hypertrophy development.

In the present study we show that AKIP1 protein levels are elevated in cardiac hypertrophy. Moreover, we show that AKIP1 overexpression can stimulate protein synthesis resulting in hypertrophy, *in vitro*.

## 2. Results

### 2.1. AKIP1 Expression Is Upregulated in Cardiac Hypertrophy/HF Models

**Figure 1:**
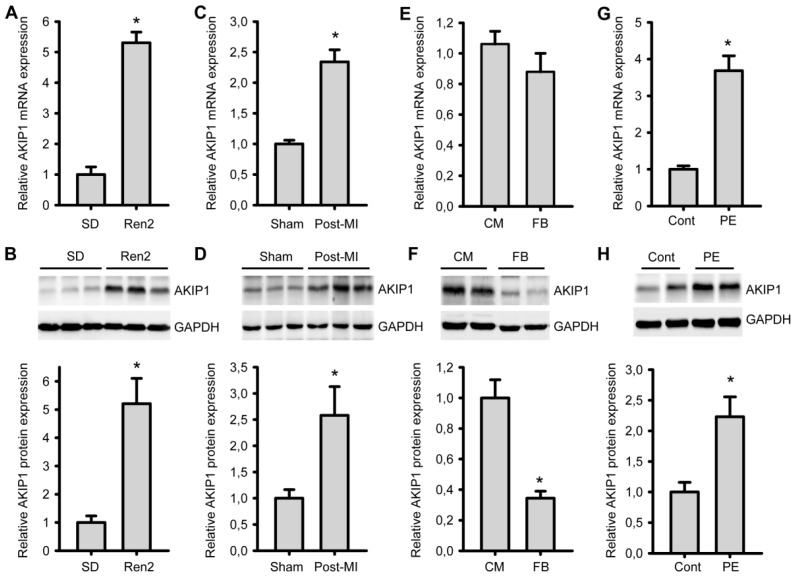
AKIP1 expression is induced in multiple cardiac hypertrophy models. (**A**) and (**B**) AKIP1 mRNA and protein expression levels were increased in Ren2 rat heart as compared to SD controls (*p < 0.01, *n* = 8); (**C**) and (**D**) AKIP1 expression was increased in post-MI rat heart as compared to sham-operated controls, at both mRNA and protein levels (*p < 0.01, *n* = 7–8); (**E**) and (**F**) AKIP1 expression in neonatal rat cardiomyocytes (CM) and fibroblasts (FB), at both mRNA and protein level (*p < 0.01, *n* = 4 and 8). mRNA and protein expression was normalized to Glyceraldehyde 3-phosphate dehydrogenase (GAPDH) for all above expression; (**G**) and (**H**) AKIP1 expression was increased in cultured neonatal cardiomyocytes stimulated with phenylephrine (PE) (50 μm, 24 h), at both mRNA and protein level. mRNA was normalized to *Cyclophilin A* and protein was normalized to GAPDH expression (*p < 0.05, *n* = 5).

In a previous gene array study, we identified *AKIP1* as a differentially expressed gene in cardiac hypertrophy [19]. By RT-PCR and by using a custom made AKIP1 specific antibody we first analyzed changes in AKIP1 expression both at the mRNA and protein level in hypertrophied and remodeled heart tissue. The functional cardiac parameters have been published before [21,22], and a summary is present in Figure S1 and Table S1. As shown in Figure 1A, AKIP1 mRNA level was significantly upregulated in hypertensive Ren2 rats, an established model of pressure overload induced hypertrophy [23], as compared to control rats. Also in cardiac tissue from rat post-MI heart failure animals, AKIP1 mRNA was significantly induced (Figure 1C). This confirms our previously published gene array data [19]. An antibody was generated to investigate AKIP1 protein expression levels. This antibody recognized recombinant AKIP1 in a dot blot (Figure S2), recognized overexpressed AKIP1 in cells (Figure S4) and the AKIP1 signal was significantly diminished after siRNA silencing (refer to Figure 3E), confirming its specificity. Importantly, Western blot analysis showed that AKIP1 protein levels were increased in both the Ren2 and the post-MI rats, as compared to their respective controls (Figure 1B,D).

We also analyzed whether the expression of AKIP1 was confined to cardiomyocytes. RT-PCR was performed on isolated primary neonatal rat cardiomyocytes and on the non-cardiomyocyte population (mostly cardiac fibroblasts). This revealed that AKIP1 expression at the mRNA levels was almost similar in both cell types, but the protein expression was clearly higher in cardiomyocytes (Figure 1E,F). This might reflect changes in processing efficiency, protein stability or turnover in these different cell types. Further investigation revealed that *AKIP1* gene expression was significantly upregulated in cultured neonatal rat cardiomyocytes treated with phenylephrine (PE), a hypertrophy inducing hormone (Figure 1G,H). In cardiac fibroblasts, no *AKIP1* upregulation was observed with PE and also TGF-β, which stimulates fibrogenesis, did not result in an induction of *AKIP1* gene expression (Figure S3). These results show that cardiac AKIP1 mRNA and protein expression is induced in several cardiac stress models and this is most likely due to increased expression in cardiomyocytes.

### 2.2. Overexpression of AKIP1 Stimulates Cardiomyocyte Hypertrophy

**Figure 2:**
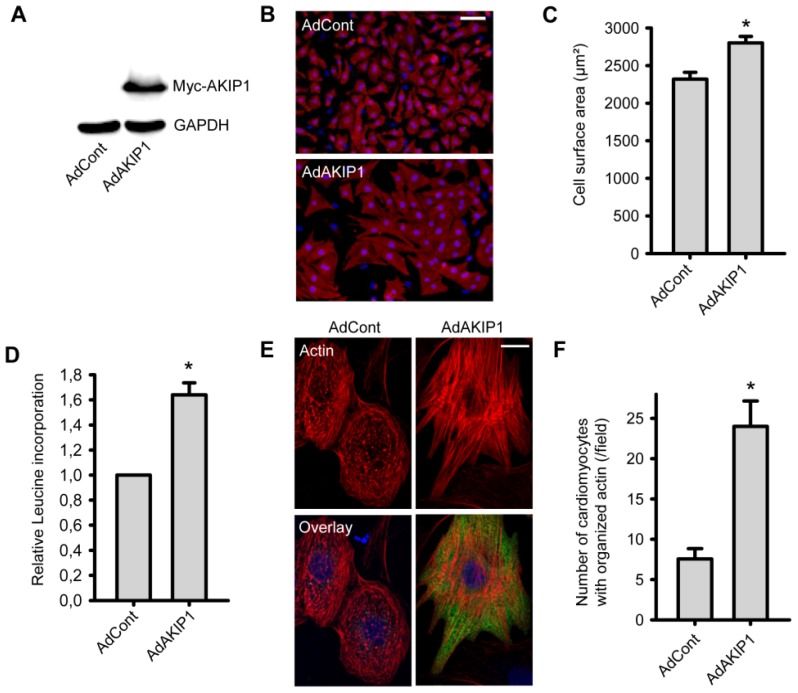
AKIP1 induces hypertrophy in neonatal cardiomyocytes. (**A**) Overexpression of myc-tagged AKIP1 protein in neonatal rat ventricular cardiomyocytes (NRVCs) for 48 h. GAPDH was used as loading control; (**B**) and (**C**) Overexpression of AKIP1 increased cell size. At the end of experiments, cells were stained with anti-α-actinin antibody (**red**) and DAPI (**blue**) and images were taken with a TissueFAXs microscope; Panel **B** shows representative pictures. Bar indicates 60 μm. Using TissueQuest software (TissueGnostics GmbH, Vienna, Austria), single cells were surrounded and the surface area per cell was calculated; Panel **C** shows the quantified cell surface area (*p < 0.05, *n* = 5 independent isolations); (**D**) Overexpression of AKIP1 increased protein synthesis. Leucine incorporation was determined as described in the Methods section (*p < 0.01, *n* = 8); (**E**) AKIP1 overexpression resulted in more organized actin fibers. Representative images of cells stained with phalloidin-rhodamine (**red**), anti-myc antibody (**green**) and DAPI (**blue**) are shown. Bar indicates 10 μm; (**F**) Quantification of the number of cells with patchy actin staining versus cells with organized actin fibers. Cells with organized actin fibers were counted in at least three fields of each well under microscope with 20× objective (*p < 0.01, *n* = 3).

Since AKIP1 expression is increased during hypertrophy development, we next aimed to evaluate whether AKIP1 modulates hypertrophic responses in NRVCs. For this purpose we generated a recombinant adenovirus expressing myc-tagged rat AKIP1. A GFP expressing adenovirus served as a control (AdCont) and overexpression of myc-AKIP1 was confirmed by Western blot analysis, using an anti-myc antibody (Figure 2A).

Using a TissueFAXs scanning microscope (TissueGnostics GmbH, Vienna, Austria) the cell surface area of more than 3000 α-actinin stained cells was automatically determined for each experiment. This revealed that AKIP1 overexpressing cells had a significant increased cell size as compared to the control group (Figure 2B,C). To further verify hypertrophy development, we also measured protein synthesis with and without AKIP1 overexpression. We observed a significant increase in protein synthesis in AdAKIP1 infected cardiomyocytes as compared to AdControl infected cells (Figure 2D). This increased protein synthesis was also dose dependent (Figure S4). We also stained infected cells with phalloidin to evaluate actin cytoskeleton organization as readout of NRVC maturation. Significant more cells with an organized cytoskeleton (aligned fibers) were observed in the AKIP1 overexpressing cells (Figure 2E,F), which is a typical sign for hypertrophied NRVCs [24].

### 2.3. AKIP1 Is Not Required for Hypertrophy Induced by Neurohormonal Signals

**Figure 3:**
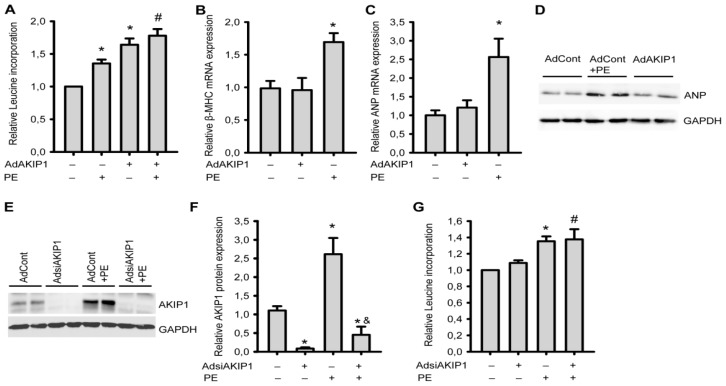
AKIP1 and neurohormonal-induced hypertrophy. One day after isolation, NRVCs were infected overnight with the indicated adenovirus in the presence of serum and subsequently serum-starved for 24 h. The cells were then stimulated with PE (50 μM) for 24 h. (**A**) AKIP1 could further increase PE-induced hypertrophy (*p < 0.01 to AdControl group, *n* = 8; # *p* < 0.01 to PE group, *n* = 8); (**B**) and (**C**) AKIP1 does not trigger the re-expression of the fetal gene program. *β-MHC* (**B**) and *ANP* (**C**) expression in control, AKIP1 overexpressing and PE treated cells are shown. mRNA expression was normalized to *Cyclophilin A* (*p < 0.01 to control, *n* = 6); (**D**) ANP protein expression in control, AKIP1 overexpressing and PE treated cells, which is consistent with mRNA expression (representative blot is shown, *n* = 3); (**E**) A representative Western blot is shown of cells treated with siAKIP1 adenovirus in the presence or absence of PE and (**F**) Quantification of all western blots (*p < 0.05 to AdControl group; & *p* < 0.05 to PE group, *n* = 3); (**G**) siAKIP1 could not inhibit PE-induced hypertrophy measured by protein synthesis (*p < 0.01 to AdControl group, *n* = 8; # *p* < 0.01 to AdsiAKIP1 group, *n* = 8).

Neurohormonal stimulation of neonatal cardiomyocytes is an established method for investigating hypertrophy *in vitro*. We therefore decided to compare AdAKIP1 induced hypertrophy with neurohormonal induced hypertrophy. Infection with AdAKIP1 resulted in stronger hypertrophy as compared to PE, and AdAKIP1 together with PE resulted in even more hypertrophy (Figure 3A). Similar results were obtained with hypertrophic stimulation using isoproterenol (Iso) and endothelin-1 (ET-1) (Figure S5).

To further investigate the AKIP1 induced hypertrophic response, we investigated the expression of several components of the fetal gene program by RT-PCR, in particular *ANP* and *β-MHC*. We observed that AKIP1 overexpression did not induce re-expression of *β-MHC* (β-myosin heavy chain) (Figure 3B) and *ANP* (atrial natriuretic peptide) (Figure 3C), in contrast to PE, which clearly induced expression of both genes. We also confirmed the absence of AKIP1 induced ANP expression at the protein level (Figure 3D).

To investigate whether downregulation of AKIP1 could prevent hypertrophy, an AdsiAKIP1 gene silencing construct was generated. We were able to reduce AKIP1 protein expression to about 10% of the endogenous levels. Also after PE treatment AKIP1 levels remained low in the siAKIP1 treated cells (Figure 3E,F). Hypertrophy development was similar in the PE treated AdsiAKIP1 cells as compared to control cells treated with PE (Figure 3G). Thus, while AKIP1 appears to potentiate hypertrophy induced by neurohormonal signals, it is not essential for neurohormonal induced hypertrophy.

### 2.4. AKIP1 Specifically Stimulates Akt Kinase and Downstream Factors Controlling Protein Synthesis

**Figure 4:**
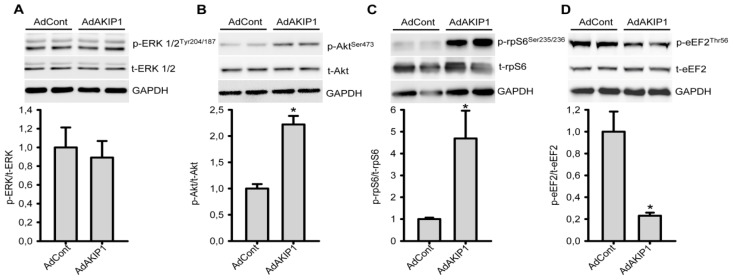
AKIP1 activates the Akt pathway and downstream proteins controlling protein synthesis. Western blot analysis of samples from AdControl and AdAKIP1 infected NRVCs after 48 h expression. The indicated proteins were detected and the prefix p- indicates the phosphorylated protein and the prefix t- indicates the total protein. GAPDH was used as a loading control. (**A**) AKIP1 overexpressing NRVCs did not induce ERK phosphorylation (*n* = 4); (**B**) Akt phosphorylation was significantly up-regulated in AKIP1 overexpressing cells (*p < 0.05, *n* = 4); (**C**) Phosphorylation of rpS6 was significantly induced in AKIP1 overexpressing cells (*p < 0.05, *n* = 4); (**D**) Less eEF2 phosphorylation was observed in AKIP1 infected cardiomyocytes (*p < 0.05, *n* = 4).

Previous studies have shown that AKIP1 is a critical modulator of cell signaling responses and we therefore hypothesized that the effects of AKIP1 on cell size and protein synthesis resulted from modulation of signaling pathways critical to hypertrophy development. Therefore, we evaluated whether AKIP1 activated nodal points in these pathways. Since AKIP1 has been shown to interact with PKA in tumor cells, AKIP1 co-immunoprecipitation was performed followed by western blot analysis of PKA. We did, however, not observe co-precipitation of PKA and AKIP1 in cardiac cells (Figure S6A). Moreover, PKA phosphorylation of the catalytic subunit (autophosphorylation) was not altered (Figure S6B) and MCIP1 expression, a marker for calcineurin/NFAT activation, remained unaltered (Figure S6C). Similarly, western blot analysis revealed that phosphorylation of ERK1/2 was not altered by AKIP1 expression in NRVCs (Figure 4A). Interestingly, Akt kinase phosphorylation was clearly increased upon AKIP1 overexpression (Figure 4B). We also analyzed ribosomal protein S6 (rpS6), a downstream target of Akt, which directly controls protein translation and cellular growth [25]. Western blot analysis of samples from control and AKIP1 overexpressing cells revealed a strong increase of phosphorylated rpS6 (Figure 4C), indicative for its activation. Another important factor controlling protein synthesis is elongation factor eEF2, which is also a target of the Akt pathway and is activated by indirect dephosphorylation [26]. Analysis revealed that phosphorylation of eEF2 was decreased in AKIP1 overexpressing cells (Figure 4D). Thus, both rpS6 and eEF2 are activated by AKIP1 and this readily explains the increased protein synthesis rate and hence hypertrophy.

IGF-1 stimulates Akt activity and hypertrophy in cardiomyocytes, we therefore wondered whether AKIP1 could play a role in IGF-1 mediated hypertrophy. Treatment of cells with IGF-1 generated a strong activation of Akt, as shown in Figure S7A. However, AKIP1 silencing did only slightly attenuate hypertrophy induced by IGF-1 (Figure S7B), indicating that AKIP1 is not a critical component of this pathway. We would like to note that ERK kinase was also activated by IGF-1. Increased ERK phosphorylation was sufficient for hypertrophy development, since Akt inhibition did not block IGF-1 induced hypertrophy (Figure S7A,C).

### 2.5. Akt Activity Is Essential for AKIP1 Induced Protein Translation and Hypertrophy Development

**Figure 5:**
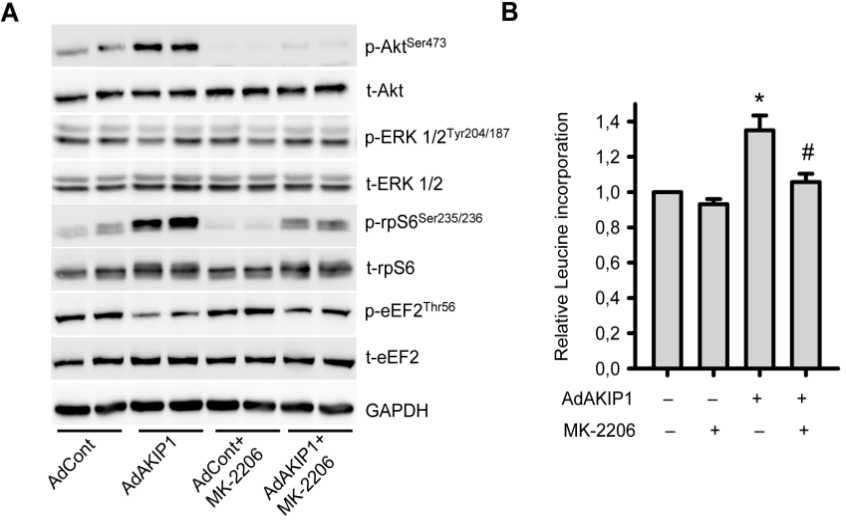
AKIP1 induced hypertrophy is Akt kinase activity dependent. (**A**) The Akt inhibitor MK-2206 (10 nM) inhibited the AKIP1-induced increase of Akt phosphorylation, but had no effect on phosphorylated ERK. Phosphorylated rpS6 and dephosphorylated eEF2 levels were attenuated by MK-2206 treatment. Cardiomyocytes were infected overnight with AdControl or AdAKIP1 followed by starvation for 24 h with or without MK-2206 treatment for 1 h (*n* = 3). Representative blots are shown; (**B**) MK-2206 inhibited AKIP1-induced hypertrophy as indicated by protein synthesis measurements. Cells were infected overnight with AdControl or AdAKIP1 followed by starvation for 48 h with or without MK-2206 treatment for 24 h (*p < 0.01 as compared to AdControl, *n* = 3; # *p* <0.05 as compared to AdAKIP1, *n* = 3).

Finally, we investigated whether Akt inhibition could prevent AKIP1 induced hypertrophy. As shown in Figure 5A, addition of the Akt specific inhibitor MK-2206, could prevent the increased phosphorylation of Akt in the presence of AKIP1. MK-2206 did not have an effect on ERK phosphorylation in NRVCs, confirming kinase specificity. Akt inhibition also prevented AKIP1 induced phosphorylation of rpS6 (Figure 5A). Dephosphorylation eEF2 was, however, only partially inhibited by Akt inhibitor, suggesting that AKIP1 does not solely control this protein via the Akt pathway. Basal protein synthesis was not affected in MK-2206 treated cells. However, the AKIP1 mediated increase in protein synthesis was totally abolished by Akt inhibition (Figure 5B). Besides MK2206, we also performed experiments with the phosphoinositide-3-kinase (PI3K) inhibitor, LY294002. As expected, LY294002 completely blocked AKIP1-induced cardiac hypertrophy (Figure S8). Together, this indicates that the PI3K/Akt pathway is essential for AKIP1 induced hypertrophy.

## 3. Discussion

AKIP1 has been mostly investigated in cancer cells, but recently its role in cardiac tissue has gained interest. AKIP1 seems to have a stress adaptive function in cardiac tissue and has recently been shown to protect the heart against ischemia/reperfusion injury [10]. Here, we extend these observations and show that AKIP1 is upregulated in the heart under conditions of cardiac wall stress, in particular during pressure overload and post-MI remodeling. Moreover, we show that AKIP1 overexpression *in vitro* triggers NRVC hypertrophy. AKIP1 was able to activate the Akt growth and survival signaling pathway and this activation was essential for the AKIP1 induced hypertrophic response. Together these data support the notion that AKIP1 is a hypertrophy responsive protein and is able to trigger adaptive pathways.

We showed that *AKIP1* gene expression is upregulated by pressure overload and post-myocardial infarction and is predominantly expressed in cardiomyocytes in the heart. Importantly, AKIP1 protein levels paralleled gene expression levels, indicating that AKIP1 is primarily regulated at the transcriptional level. Although, we only looked at pathological stress models, increased *AKIP1* gene expression has also been reported in a gene expression study on left ventricle tissue of moderately exercised rats [27]. Thus AKIP1 expression is also induced by physiological stress. Moreover, AKIP1 expression is induced by oxidative stress and during ischemia/reperfusion injury [10], suggesting that increasing AKIP1 expression represents a general adaptation to multiple stressors.

One of the adaptations to cardiac stress is cardiomyocyte hypertrophy and we could show here that AKIP1 can induce a hypertrophic response in NRVCs. We have also tested a number of other genes identified in our previous screen [19], like Dhrs7c [28], but none of these induced hypertrophy, suggesting specificity. Several hypertrophic signaling pathways were analyzed, but only the Akt pathway, which has a general function in cell survival and growth [29,30], was activated by AKIP1. Activation of Akt can be mediated by hormones [31], cytokines of the IL-6 family [32] and other plasma proteins like periostin, which are all elevated during cardiac stress [19,32]. In contrast to these factors, AKIP1 is not an extracellular protein and hence is unlikely to signal via membrane receptors, but probably modulates Akt activity via intracellular interactions. A number of other intracellular factors have been identified as modulators of Akt activity, including CTMP, Trb3, Hsp27 and stress-activated kinases of the JNK family [30,33]. Hence, it is not unlikely that AKIP1 might act together with or via these intracellular modulators. This will require further investigations.

Although, AKIP1 expression was induced by PE, concomitant silencing of AKIP1 did not prevent PE induced hypertrophy *in vitro*. We cannot exclude that 10% residual AKIP1 was still sufficient for PE induced hypertrophy and that full knock out of AKIP1 may prevent PE induced hypertrophy. We like to note, however, that PE induces multiple signaling pathways, including the ERK pathway and calcium signaling [34,35] and hence it can be readily envisioned that depletion of a single factor (AKIP1) may not prevent PE induced hypertrophy. These pathways also trigger fetal gene expression, which is absent in AKIP1 overexpressed cells. AKIP1 appeared to potentiate neurohormonal induced hypertrophy and therefore seems to have a modulating, rather than essential, role in PE induced hypertrophic responses *in vitro*.

Although Akt activation has been predominantly linked to physiological hypertrophy, based on mouse knock-out and overexpression studies, long term overexpression also resulted in pathological hypertrophy. Moreover, rodent studies have shown cardiac Akt activation by different stress factors including pressure overload and hypoxia [33,36] and also in HF patients Akt is activated [37]. Considering the connection with Akt, it is likely that increased AKIP1 levels are an adaptive stress response, but it cannot be excluded that long term AKIP1 expression may finally lead to maladaptive actions. Whether Akt is also involved in AKIP1 mediated ischemia/reperfusion protection is not known, but considering the role of Akt in cell survival this may well be the case. AKIP1 has also been linked to apoptotic factors [10,15] and it is therefore not unlikely that AKIP1 conveys stress adaptation via multiple mechanisms.

## 4. Materials and Methods

### 4.1. Isolation and Culturing of Primary Cardiomyocytes

Neonatal rat ventricular cardiomyocytes (NRVCs) were isolated from 1–3 days old neonatal rats, as previously described [38,39]. NRVCs were grown in DMEM (Sigma D5671, St. Louis, MO, USA) supplemented with 5% fetal calf serum (FCS: Sigma F9665, St. Louis, MO, USA) and penicillin-streptomycin (100 U/mL-100 μg/mL) (Sigma P0781, St. Louis, MO, USA). For adenoviral infections, NRVCs were infected with adenovirus (MOI 10–25) one day after isolation, in medium with 5% FCS, and starved the next day for 24 h, like for the non- or control infected cells. The used MOI’s gave similar results. To induce hypertrophy, after starvation for 24 h, cells were treated with phenylephrine (PE) (50 μM) for another 24 h.

### 4.2. Animal Studies

All animal studies were conducted in accordance with the NIH Guide for the Care and Use of Laboratory Animals and were submitted to, and approved by, the Committee for Animal Experiments of the University of Groningen. Ren2 transgenic rats, overexpressing the mouse renin gene, were obtained from the Max Delbrück Center for Molecular Medicine (Berlin-Buch, Berlin, Germany). Cardiac hypertrophy and heart failure development in Ren2 rats has been described before [23,40]. Cardiac samples from Ren2 and Sprague Dawley rats, the genetic background of the Ren2 rats, were used for RNA analysis as described in previous studies [21,23]. Cardiac samples of sham and post-myocardial infracted (post-MI) rats used here were from a previously described study [22]. In short, left coronary artery ligation surgery was performed and as a control, animals were sham-operated. Twelve weeks after operations animals were sacrificed and cardiac tissue was collected.

### 4.3. Generation and Purification of Antibody

A cDNA fragment of mouse *AKIP1* encoding amino acids 1–142 was subcloned into the *Escherichia coli* expression vector pQE-80. His6 tagged recombinant AKIP1 fragment was produced in the BL21 (RIL) *E. coli* strain (Agilent Technologies, Santa Clara, CA, USA) and cells were lysed by sonication in T10N50E1 (10 mM Tris, pH 8.0, 50 mM NaCl, 1 mM EDTA) buffer including proteinase inhibitors cocktail (Roche, 11873580001, Penzberg, Germany). The protein pellet, containing AKIP1 inclusion bodies was washed once with T10N50E1 buffer and dissolved in 6M Guantinine buffer (pH 8.0). His-tagged AKIP1 protein was subsequently purified using Ni-NTA Agarose according to the manufacturer instructions (Qiagen, Venlo, The Netherlands). Rabbit polyclonal antibody against recombinant AKIP1 was generated by Biogenes (Berlin, Germany). Specific AKIP1 antibody was purified using the same recombinant protein immobilized on affi-gel 10 beads (Bio-Rad, Hercules, CA, USA).

### 4.4. Generation of Recombinant Adenovirus and Transient Infection

Adenoviral constructs were generated with the ViraPower^™^ adenoviral expression system from Invitrogen (Carlsbad, CA, USA). For *AKIP1* cloning, total rat heart cDNA was used and *AKIP1* cDNA was amplified by polymerase chain reaction (PCR) using the forward and reverse primers listed in Table S2. The PCR product was cloned directly behind a CMV promoter. For a *N*-terminal myc-tagged AKIP1, this sequence was cloned in frame with a triple myc-tag sequence. Cloning was verified by sequencing and the cDNA sequence was identical to the rat *AKIP1* deposited GenBank sequence NM_001108497. For adenoviral-siAKIP1, the specific siRNA oligos listed in Table S2 against the rat *AKIP1* gene were used. The annealed primers were cloned into a pENTR4 vector containing a H1-promoter and a GFP marker gene. Recombinant adenovirus was generated as previously described [28]. For control infections a corresponding GFP expressing adenovirus was used.

### 4.5. Real Time PCR

Total RNA was isolated either using TRIzol reagent (Life Technologies, Carlsbad, CA, USA) or using a kit (Bioke, Leiden, The Netherlands) and cDNA was synthesized using Reverse Transcriptional kit (Qiagen, Venlo, The Netherlands) following manufactures instructions. Relative gene expression was determined by quantitative real time PCR (RT-PCR) on a Bio-Rad CFX384 real time system using SYBR Green dye. Gene expressions were corrected for reference gene values (*Cyclophilin A* or *GAPDH*), and the calculated values were expressed relative to the control group per experiment. Primer sequences are listed in Table S3.

### 4.6. Protein Synthesis Assay

Cells were grown in 12-well plates and subsequently infected and then starved for 24 h in DMEM containing starvation medium lacking FCS. Cells were cultured for an additional 24 h and leucine incorporation was determined as described before [38].

### 4.7. Immunofluorescence (IF) and Cell Size Measurement

NRVCs were plated in 12 well plates with 18 mm coverslips coated with laminin (Millipore, Billerica, MA, USA). 72 h after infection cells were washed once with PBS and fixed 10 min with paraformaldehyde buffer at 4 °C, followed by permeabilization with 0.5% Triton X100 for 5 min. Cells were subsequently incubated with monoclonal anti-α-actinin antibody (Sigma, St. Louis, MO, USA) or a monoclonal 9E10 anti-myc antibody diluted in 1% BSA + PBS for 1 h at RT, after washing, cells were incubated with Alexa 555 donkey anti-mouse secondary antibody (Invitrogen, Carlsbad, CA, USA) or Alexa 488 goat anti-mouse secondary antibody (Invitrogen, Carlsbad, CA, USA) and/or with palloidin-rhodamine (Invitrogen, Carlsbad, CA, USA) and DNA was counterstained with Tropo3 (Invitrogen, Carlsbad, CA, USA) or DAPI. Coverslips were mounted with mounting medium and slides were imaged using a Confocal microscope (SP2 AOBS, Leica, Wetzlar, Germany) or a TissueFAXs (TissueGnostics GmbH, Vienna, Austria). The latter was used for cell size determination, together with TissueQuest fluorescence analysis software. This allows automatic counting of single cells and the software can surround cells and determine the surface area per cell. For quantifying cells with organized actin fibers, a blinded investigator counted cells manually and distinguished between patchy staining and fiber staining.

### 4.8. Western Blot

Protein was isolated in RIPA buffer (50 mM Tris pH 8.0, 1% nonidet P40, 0.5% deoxycholate, 0.1% SDS, 150 mM NaCl) supplemented with 10 μL/mL phosphatase inhibitor cocktail 3 (Sigma p2850, St. Louis, MO, USA), protease inhibitor cocktail (Roche, 11873580001, Penzberg, Germany) and 1 mM phenylmethylsulfonyl fluoride (PMSF) (Roche,10837091001, Penzberg, Germany). Protein concentration was determined with a DC protein assay kit (Bio-Rad, Hercules, CA, USA). Equal amounts of proteins were separated by SDS-PAGE and proteins were transferred on PVDF or nitrocellulose membranes. For detection of specific proteins the following antibodies were used: anti-phosphorylated-Akt^Ser473^, anti-total-Akt, anti-total-ERK1/2, anti-phosphorylated-rpS6^Ser235/236^, anti-total-rpS6, anti-phosphorylated-eEF2^Thr56^ and anti-total eEF2 monoclonal antibodies. All from Cell Signaling Technology and used at a dilution of 1:1000. Anti-phosphorylated-ERK1/2^Tyr204/187^ monoclonal antibody (1:1000) and anti-ANP antibody (1:500) were from SantaCruz (Dallas, TX, USA). Monoclonal anti-GAPDH antibody (Fitzgerald, Acton, MA, USA) or anti-tubulin antibody (Sigma, St. Louis, MO, USA) was used as a loading control. After incubation with secondary antibodies signals were visualized with ECL and analyzed with densitometry (Syngene, Cambridge, UK).

### 4.9. Statistical Analysis

All values are presented as means ± standard errors of the mean (SEM). Independent-samples t-test was performed to compare the difference between two groups. Comparison between more than two groups, data was assessed by One-Way Anova followed by *post hoc* Tukey test. A value of *p* < 0.05 was considered to be significant. SPSS software (PASW Statistics 18, IBM Corporation, Armonk, NY, USA) was used in the statistics analysis.

## 5. Conclusions

In conclusion, our findings demonstrate that AKIP1 is a cardiomyocyte expressed protein that is upregulated during hypertrophy inducing conditions. AKIP1 is able to stimulate protein synthesis and this requires Akt activity. Accumulating evidence indicates now that AKIP1 is a stress responsive gene in the heart and may have an adaptive function against cardiac stress.

## Supplementary Information
