# Supplementary material for: AKIP1, a Cardiac Hypertrophy Induced Protein that Stimulates Cardiomyocyte Growth via the Akt Pathway
Source: Int J Mol Sci. 2013 Oct 28;14(11):21378–93. doi: 10.3390/ijms141121378 (PMC3856010; doi:10.3390/ijms141121378)
Supplement: Supplementary file 1 [file ijms-14-21378-s001.pdf]

Supplementary Information

Figure S1. (**A**) *ANP* gene expression was significantly upregulated in Ren2 rat hearts as compared to SD rat hearts (* $p < 0.01$ compared to SD group, $n = 8$); (**B**) *ANP* gene expression was significantly upregulated in post-MI rat hearts as compared to sham control rat hearts (* $p < 0.01$, $n = 7–8$). mRNA expression was normalized to *GAPDH* for both experiments.

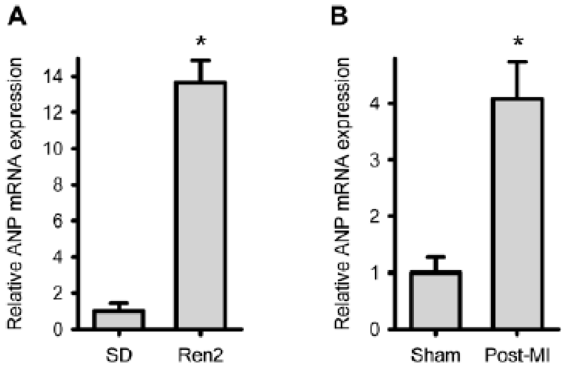

Figure S2. Specificity of anti-AKIP1 antibody was confirmed by dot blot. 25 ng of recombinant full-length AKIP1 or BSA was dropped on nitrocellulose membranes and the membranes were blocked with 5% milk followed by incubation with anti-AKIP1 antibody. Different dilutions of antibody were used to confirm the specificity.

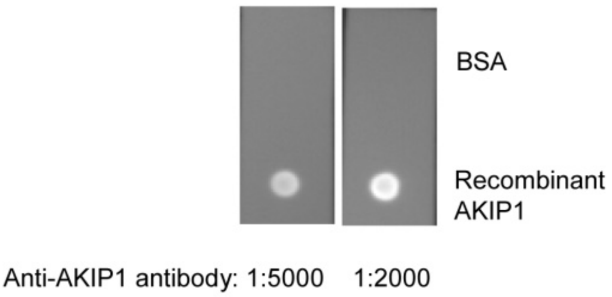

Figure S3. *AKIP1* gene expression does not change in cultured cardiac fibroblasts after stimulation with different agents. Cultured neonatal rat cardiac fibroblasts were starved for 24 h and stimulated with PE (50 μM) or TGF-β (5 ng/mL) for 24 h. *AKIP1* mRNA expression in these cells was determined by RT-PCR and expression was normalized to *GAPDH*. No significant differences were observed ($n = 4$).

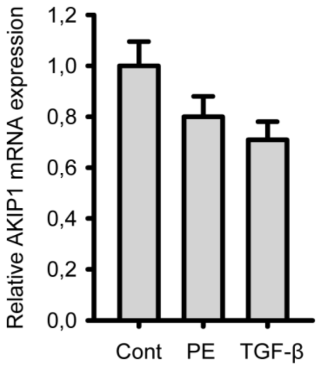

Figure S4. AKIP1-induced hypertrophy in cardiomyocytes is dose dependent. Cells were infected overnight by control or AKIP1 adenovirus at different MOI (from 1 to 10) followed by 48 h starvation. Protein was detected by anti-AKIP1 antibody and representative blot is shown. Tubulin was used as loading control. Hypertrophy effect was measured by [3H]-leucine incorporation, which reveals a dose dependent effect (* $p < 0.05$ as compared to AdControl group, $n = 6$).

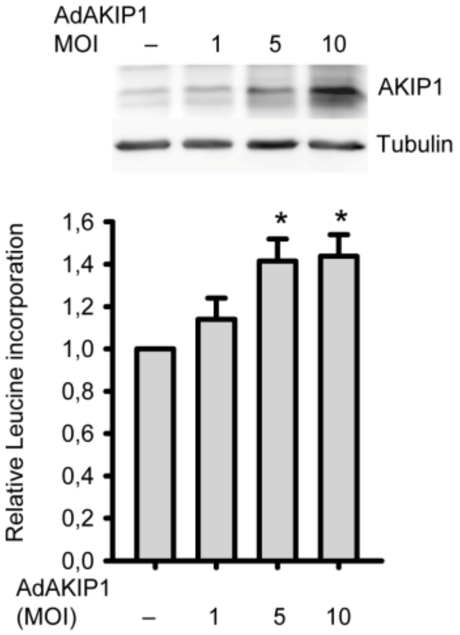

Figure S5. AKIP1 can further increase ET-1- and Iso-induced cardiac hypertrophy. Cells were infected overnight with adenovirus followed by 48 h starvation with or without ET-1 (10 nM) or Iso (10 μM) treatment for 24 h (* $p < 0.01$ as compared with AdControl group, $n = 8$; # $p < 0.01$ compared with ET-1 or Iso group, $n = 8$). (**A**)AKIP1 could further increase ET-1 induced cardiac hypertrophy; (**B**) AKIP1 could further increase Iso induced cardiac hypertrophy.

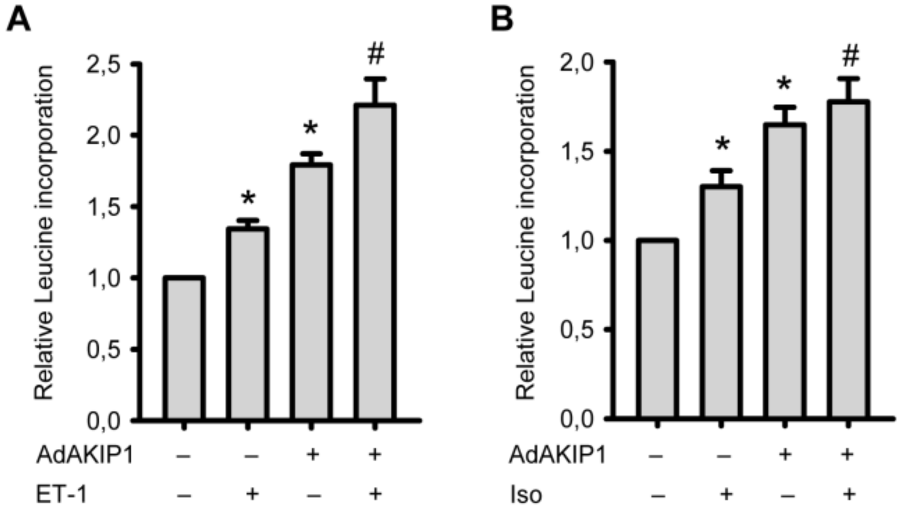

Figure S6. (**A**) AKIP1 did not co-immunoprecipitate with PKA in cardiac cells. Cardiac HL-1 cells were infected with myc-AKIP1 adenoviruses for 24 h, followed by 24 h starvation. Cell lysates were incubated with myc-specific antibodies on protein G beads (Santa Cruz) at 4 °C overnight. After extensive washing with PBS, the beads were boiled in sample buffer and subjected to western blotting with anti-PKA (Cell Signaling) or anti-myc 9E10 antibody; (**B**) AKIP1 did not activate PKA. Cells were infected overnight with control or myc-AKIP1 adenovirus followed by 24 h of starvation. Total protein (20 μg/lane) was separated by SDS-PAGE and subjected to immunoblotting for phospho- and total PKA protein (anti- phosphorylated-PKA^{Thr197} and anti-total-PKA antibodies were from cell signaling and used as a dilution of 1:1000). GAPDH was used as loading control. Representative blots are shown (n = 3); (**C**) AKIP1 overexpression did not induce *MCIP1* gene expression in NRVCs. Gene expression was normalized to *Cyclophilin A* expression (n = 5).

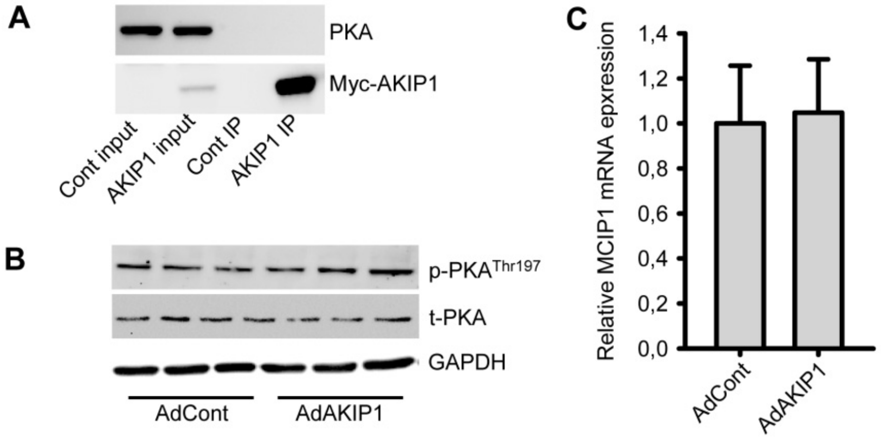

Figure S7. (**A**) Akt phosphorylation was strongly induced by IGF-1 (10 nM, 5 mins) and ERK phosphorylation was induced by both IGF-1 (10 nM, 5 mins) and PE (50 μM, 5 mins) in NRVCs; (**B**) siAKIP1 could not block IGF-1 induced hypertrophy as measured by leucine incorporation. Cells were infected overnight with AdControl or AdAKIP1 adenovirus, followed by 48 h starvation with or without IGF-1 (10 nM) for 24 h (* $p < 0.05$ compared to AdControl group, n = 5); (**C**) Akt inhibitor, MK-2206, did not inhibit IGF-1 induced cardiac hypertrophy. Cells were stimulated as above, MK-2206 (10 nM) was added in indicated wells 30 mins before IGF-1 (* p <0.05 compared to AdControl group, n = 3).

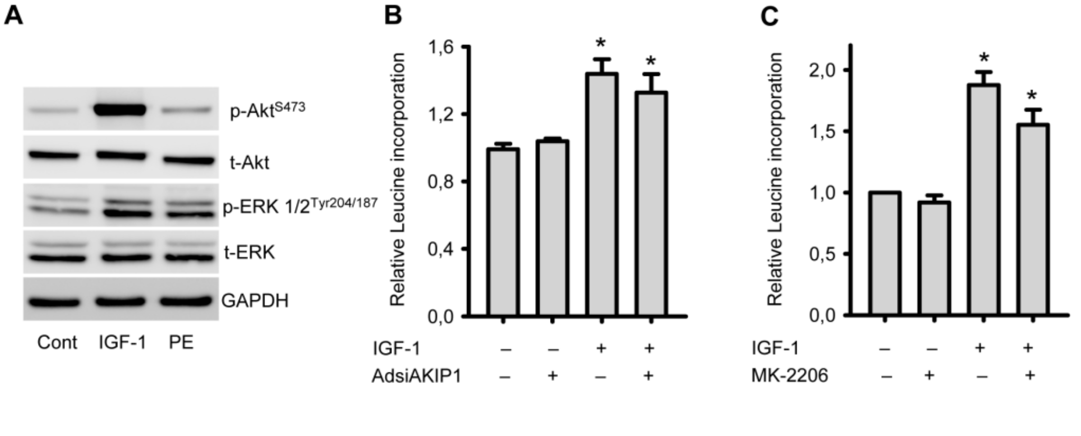

Figure S8. Inhibition of PI3K with LY294002 (10 µm) could fully block AKIP1 induced hypertrophy as measured by protein synthesis. Cells were infected overnight with AdControl or AdAKIP1 followed by starvation for 48 h with or without LY294004 treatment for 24 h (* $p < 0.05$ compared to AdControl group; # $p < 0.05$ compared to AdAKIP1 group, $n = 3$).

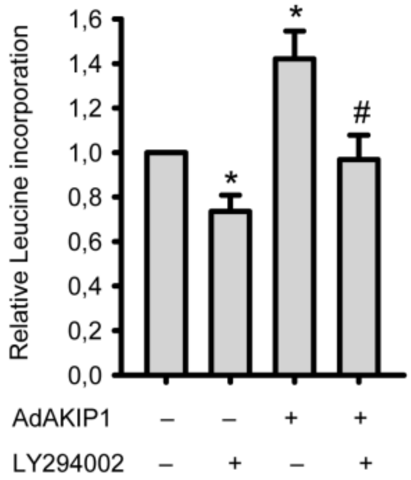

Table S1. Baseline characteristics of rat HF models.

	SD	REN2	MI-sham	MI
n	12	12	9	12
SBP, mmHg	120 ± 4	112 ± 8	114 ± 14	108 ± 4
DBP, mmHg	83 ± 2	78 ± 6	88 ± 3	78 ± 4
HR, beats/min	352 ± 17	347 ± 12	300 ± 17	274 ± 11
LVEDP, mmHg	5 ± 1	10 ± 1 *	13 ± 3	22 ± 1 #
LVESP, mmHg	121 ± 3	100 ± 5	125 ± 6	108 ± 4 #
dPdtmax, mmHg/s	7993 ± 290	5625 ± 356 *	11847 ± 761	9180 ± 537 #
dPdtmin, mmHg/s	−9211 ± 666	−5730 ± 335 *	−10368 ± 800	−7311 ± 595 #
HW/BW, mg/g	3.3 ± 0.1	5.2 ± 0.1 *	3.0 ± 0.9	4.0 ± 0.7 #
BW, g	367 ± 6	309 ± 7 *	293 ± 5	281 ± 5

Summary of functional cardiac parameters of animals used in this study. The full studies have been published before [21,22]. Data are presented as means ± SE. HF, heart failure; SD, Sprague-Dawley; MI, myocardial infarction; SBP, systolic blood pressure; DBP, diastolic blood pressure; HR, heart rate; LVEDP, left ventricular end diastolic pressure; LVESP, left ventricular end systolic pressure; dPdtmax and dPdtmin are indexes of maximal contraction and relaxation; HW, heart weight; BW, body weight. *Values were significantly different ($p < 0.05$) from SD; #values were significantly different ($P < 0.05$) from sham.

Table S2. Primers used for cloning.

Primers	5'-3'
AKIP1-foward	GAAGGATCCGTCGACATGGAATACTGCCTGGCGGC
AKIP1-reverse	GAACTCGAGTCATACGGGGAACACCAAGTCCAC
siAKIP1-forward	GATCCCGTGGTTGCAGTTGACTCGTTCAAGAGAGACCGAGTC AACTGCAACCACTTTTTGGAAA
siAKIP1-reverse	AGCTTTTCCAAAAAGTGGTTGCAGTTGACTCGGTCTCTTGAAC CGAGTCAACTGCAACCAACGG

Table S3. Primers used for Real-Time PCR

Genes	5'-3' forward	5'-3' reverse
GAPDH	CATCAAGAAGGTGGTGAAGCGC	ACCACCCTGTTGCTGTAG
CyclophilinA	CAGATCGAGGGATCGATTCAG	TCACCACTTGACACCCTCATTC
AKIP1	TGGTCCAGGAAGCATCTATC	CAACCACATGCGTCTTCTTG
ANP	ATGGGCTCCTTCTCCATCAC	TCTACCGGCATCTTCTCCTC
β-MHC	GTCAAGCTCCTAAGTAATCTGTT	GAAAGGATGAGCCTTTCTTTGC
MCIP1	AGCGAAAGTGAGACCAGGGC	GGCAGGGGGAGAGATGAGAA

© 2013 by the authors; licensee MDPI, Basel, Switzerland. This article is an open access article distributed under the terms and conditions of the Creative Commons Attribution license (http://creativecommons.org/licenses/by/3.0/).
